# Prediction of the Atomization Process in Respimat^®^ Soft Mist^TM^ Inhalers Using a Volume of Fluid-to-Discrete Phase Model

**DOI:** 10.3390/bioengineering12030264

**Published:** 2025-03-06

**Authors:** Ted Sperry, Yu Feng

**Affiliations:** School of Chemical Engineering, Oklahoma State University, Stillwater, OK 74078, USA; ted.sperry@okstate.edu

**Keywords:** Soft Mist inhaler (SMI), volume of fluid-to-discrete phase model (VOF-to-DPM), atomization, emitted droplet size distribution

## Abstract

This study investigates the atomization process in Respimat^®^ Soft Mist^TM^ Inhalers (SMIs) using a validated Volume of Fluid (VOF)-to-Discrete Phase Model (DPM) to simulate the transition from colliding liquid jets to aerosolized droplets. Key parameters, including colliding jet inlet velocity, surface tension, and liquid viscosity, were systematically varied to analyze their impact on the atomization, i.e., aerosolized droplet size distributions. The VOF-to-DPM simulation results indicate that higher jet inlet velocities enhance ligament fragmentation, producing finer and more uniform droplets while reducing total atomized droplet mass. The relationship between surface tension and atomization performance in colliding jet atomization is not monotonic. Reducing surface tension plays a complex dual role in the atomization process. On the one hand, lower surface tension enhances the likelihood of liquid jet breakup into a liquid sheet, leading to the formation of smaller ligaments under the same airflow conditions and shear forces. This increases the probability of generating more secondary droplets. On the other hand, reduced surface tension also destabilizes the liquid surface shape, decreasing the formation of fine, high-sphericity droplets in regimes where surface tension is a dominant force. Viscosity also influences atomization through complex mechanisms, i.e., lower viscosity reduces resistance to ligament breakup but promotes droplet interactions and coalescence, while higher viscosity suppresses ligament fragmentation, generating larger droplets and reducing atomization efficiency. The validated VOF-to-DPM framework provides critical insights for enhancing the performance and efficiency of inhalation therapies. Future work will incorporate nozzle geometry, jet impingement angles, and surfactant effects to better understand and optimize the atomization process in SMIs, focusing on achieving preferred droplet size distributions and emitted doses for enhanced drug delivery efficiency in human respiratory systems.

## 1. Introduction

Inhaled aerosolized medications are a cornerstone of respiratory disease treatment, with conditions such as chronic obstructive pulmonary disease (COPD) affecting over 250 million people globally and ranking as the third leading cause of death worldwide [1,2]. Despite their widespread use, current inhalation therapies, particularly dry powder inhalers (DPIs) and pressurized metered-dose inhalers (pMDIs), suffer from inefficient drug delivery [3]. This inefficiency is primarily due to high-plume momentum in pMDIs, leading to premature deposition in the mouth–throat region via inertial impaction, reducing the dose available for deep lung delivery [4].

To address these challenges, Soft Mist inhalers (SMIs), such as Respimat^®^ SMI (Boehringer Ingelheim International GmbH, Ingelheim am Rhein, Germany), were developed as a propellant-free atomization alternative to traditional pMDIs. The key benefits of SMIs include (1) lower plume velocity, which reduces inertial impaction and increases lung deposition; (2) higher fine particle fraction, improving drug delivery efficiency to smaller airways; (3) propellant-free design, complying with environmental regulations set by the Montreal Protocol [5]; and (4) more compatibility with inspiratory effort, making it more suitable for patients with limited inhalation capacity. These advantages make Respimat^®^ SMI a promising solution for improving medication delivery to targeted lung regions, particularly in diseases such as COPD.

**Figure 1:**
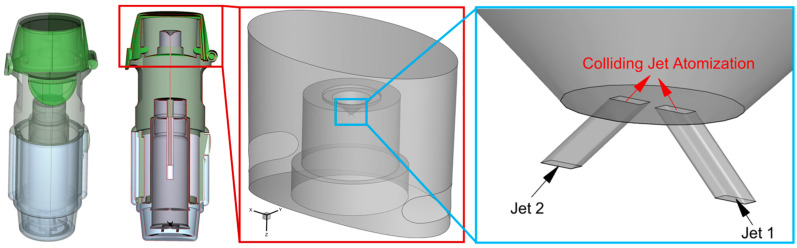
Respimat^®^ Soft Mist inhaler (SMI) with details of the colliding jets setup.

Specifically, Respimat^®^ SMI utilizes impinging jets to produce inhalable therapeutic aerosols with fine droplet distributions (see Figure 1). Employing such a relatively novel atomization method, SMIs are propellant-free and potentially have advantages mentioned in the last paragraph that can potentially enhance the delivery of medications to small airways, especially for patients with limited inhalation capabilities [6]. These advantages include the high fine particulate matter fraction, low-plume velocity, and an atomization method that does not depend on respiratory actuation. Specifically, the smaller droplet sizes and lower initial spray velocity produced by the atomization in SMI avoid this unwanted deposition in the mouth-to-throat region.

However, the development and finalization of Respimat^®^ SMI design, especially the colliding jets, largely rely on benchtop experiments [4]. Therefore, it will be beneficial to develop a first principle-based digital twin system that can accurately and explicitly predict the atomization process since it will allow for the improved development of similar devices in the future that can generate preferred droplet size distributions for better drug delivery to designated lung sites for specific diseases in a non-invasive, cost-effective, and time-saving manner. Specifically, computational fluid dynamic (CFD) models, i.e., the VOF-to-DPM approach [7,8,9,10,11], have been proven to be able to simulate and visualize multiple types of atomization processes, capturing the transition from liquid jets to aerosolized droplets under varying design and operating conditions, which are challenging to capture experimentally.

There have been a limited number of VOF-to-DPM efforts to model SMIs focusing on simulating the atomization process. Early studies employed simplified geometries and 2D models to predict spray characteristics, such as droplet size distribution, spray angles, and velocity patterns. For example, Ge et al. [12] modeled the impinging jet atomization in SMIs using a 2D VOF model, demonstrating the influence of nozzle geometry and jet collision angle on spray dynamics. Jin et al. [7] utilized a 3D VOF-to-DPM approach to study the atomization process, incorporating nozzle geometry and validating their results against experimental data.

Despite advancements in numerical modeling, existing efforts face significant limitations. Many studies need to be more accurate in the geometry of the atomization chamber, which reduces their applicability to real-world designs. Surface tension and liquid viscosity, which are critical factors in droplet formation, often rely on approximations that may only partially capture the complexities of high-curvature interfaces. These deficiencies hinder the development of optimized SMI designs and prevent a comprehensive understanding of the interplay between nozzle design, drug formulation, spray dynamics, and the key metrics for SMI performance, which is the emitted droplet size distributions.

Therefore, to address the gaps mentioned above, this study developed a validated 3D VOF-to-DPM to explicitly simulate the atomization process in Respimat^®^ SMI, from the collision of liquid jets to the generation of therapeutic aerosolized droplets. This study incorporates realistic nozzle geometries, explicit surface tension models, and multiscale droplet formation dynamics. Additionally, parametric analysis provides insights into how colliding jet inlet velocity, surface tension, and viscosity can influence the atomization process and the resulting emitted droplet size distributions. This VOF-to-DPM and simulations aim to contribute to the future development of improved SMI designs for more efficient pulmonary drug delivery in a non-invasive, cost-effective, and time-saving manner.

## 2. Materials and Methods

### 2.1. Geometry and Mesh

**Figure 2:**
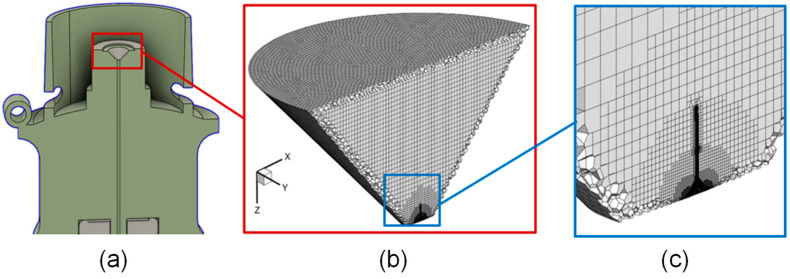
Mesh details in the atomization region of Respimat^®^ SMI: (**a**) SMI flow channel structure at the midplane; (**b**) adaptive mesh at the atomization region; and (**c**) mesh near the colliding jets.

The geometry employed in this study represents the Respimat ^®^ SMI in Figure 1 and Figure 2, with a conical fluid region within the inhaler mouthpiece pointing toward the user. The liquid jets are spaced 50 µm apart and angled at $45^{\circ }$ to provide a $90^{\circ }$ impingement angle. These ducts are modeled at 8 µm wide and 12 µm long to represent the square cross-section of the nozzle inlet into the fluid domain. Rather than simulating the ducts, the velocity profile and direction are defined at the rectangular inlets (see Figure 1 and Figure 2).

A dynamic meshing algorithm, often referred to as adaptive mesh refinement (AMR), was employed in mesh generation. The selected initial mesh for this zone contained approximately 400,000 polyhedral and hexahedral elements. This cell count increased upon introducing the liquid jets as adaptive refinement subdivides the elements surrounding the air–liquid interface. Refinement and coarsening for these cases are defined by the volume fraction gradient in each cell, ensuring detailed tracking of the boundary where air meets the liquid jet. This refinement is performed at each flow time step to ensure the moving interface is resolved at all points. Upon the transition of resolved VOF droplets into the discrete phase, the mesh refinement in that location is reverted to the original mesh to reduce computational cost. Further details are provided in Section 2.2.

### 2.2. Governing Equations

#### 2.2.1. VOF Model

The following governing equations represent the primary and secondary continuous phases (i.e., air and liquid solution) of the VOF model. The VOF model tracks the volume fraction of each phase in the mesh cells and calculates the interface between air and liquid in this study. To track the secondary phase (i.e., liquid solution) and the eventual transition into a discrete phase, a volume fraction $\alpha_{i}$ for each phase, in this case for a liquid and gas phase, is defined. Specifically, $\alpha_{i}$ should satisfy the following:

(1) $$\sum_{i=1}^{2}\alpha_{i}=1$$

where i=1 is the primary phase (i.e., air) and i=2 is the secondary phase (i.e., liquid solution). α_i_ = 1 indicates either phase or the cell contains only one phase. Interface-containing cells, where 0 < *α_i_* < 1, are used to reconstruct the free fluid surface and comprise both fluids. Accordingly, the volume-averaged density ρ and viscosity μ in each mesh cell can be calculated using the following:

(2) $$\rho =\sum_{i=1}^{2}\left(\alpha_{i}\rho_{i}\right)$$

(3) $$\mu =\sum_{i=1}^{2}\left(\alpha_{i}\mu_{i}\right)$$

Using Equations (1)–(3), mass conservation for a system of two immiscible fluids can be given as follows:

(4) $$\frac{\partial \rho }{\partial t}+\frac{\partial \left(\rho u_{i}\right)}{\partial x_{i}}=0$$

where $u_{i}$ is the fluid velocity along direction $x_{i}$.

Additionally, the momentum equation can be given as follows:

(5) $$\frac{\partial \left(\rho u_{i}\right)}{\partial t}+\frac{\partial \left(\rho u_{i}u_{j}\right)}{\partial x_{j}}=-\frac{\partial p}{\partial x_{i}}+\frac{\partial \tau_{ij}}{\partial x_{j}}+\rho g_{i}+F_{\sigma ,i}$$

Here, ρ represents the fluid density within the cell based on Equation (2), and the viscous stress tensor $\tau_{ij}$ is expressed as follows:

(6) $$\tau_{ij}=\mu \left[\left(\frac{\partial u_{i}}{\partial x_{j}}+\frac{\partial u_{j}}{\partial x_{i}}\right)-\frac{2}{3}\delta_{ij}\frac{\partial u_{k}}{\partial x_{k}}\right]$$

The final term in Equation (5), $F_{\sigma ,i}$, represents the continuum surface tension force, which is calculated here by the following equations as proposed by Brackbill et al. [13], i.e.,

(7) $$F_{\sigma ,i}=\sigma \kappa n_{i}\delta_{s}$$

(8) κ=∇⋅n

The surface tension coefficient, *σ*, is multiplied by the interface curvature *κ*, the local interface average direction ***n***, and the Dirac function $\delta_{s}$ which ensures that the force only acts upon the fluid interface. Equation (8) shows how the curvature *κ* is calculated from the divergence of the interface normal vector ***n***, which describes the orientation of the interface in each interface-containing cell. Specifically, n is defined as the following:

(9) $$n=\nabla \alpha_{2}$$

Here, $\nabla \alpha_{2}$ is numerically calculated using the piecewise linear interface reconstruction (PLIC) method [14].

#### 2.2.2. VOF-to-DPM Transition

When a droplet of the secondary phase (i.e., liquid solution) detaches from the breakup region where the jets collide and liquid sheets form, it is considered to convert from the VOF domain into the DPM tracking method (i.e., droplets will be calculated as spheres with constant diameters). These droplets form from highly irregular ligaments during droplet formation and secondary breakup, so filtering only the detached and spherical droplets is essential. This conversion occurs when the diameter of this lump is within a specified range, and the sphericity reaches a similar criterion. Determining whether a droplet meets the criteria for being considered spherical involves two main parameters, i.e., (1) the cutoff diameter ranges for droplets and (2) the sphericity of the droplet. Specifically, the VOF-to-DPM method quantifies the sphericity of a detached liquid lump using asphericity (A), which is defined by the following:

(10) $$A=1-n\cdot \frac{r}{\left|r\right|}$$

**Figure 3:**
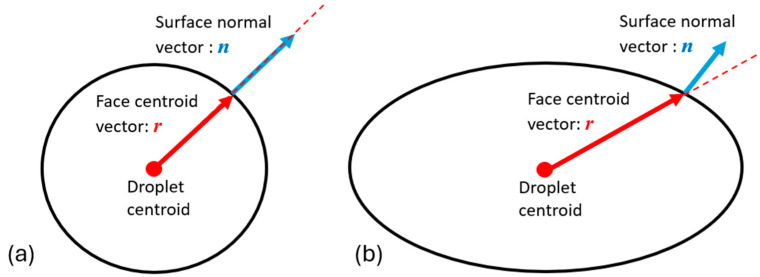
Illustration of droplet asphericity based on vector alignment: (**a**) spherical droplet with asphericity A=0, where the surface normal vector n aligns with the face centroid vector r, and (**b**) droplet with asphericity A > 0, showing a deviation in vector alignment due to shape deformation.

Specifically, A quantifies how much a detached liquid shape deviates from being perfectly spherical by comparing the alignment of the surface normal (***n***) with the normalized radius vector (***r***), which is shown in Figure 3.

As shown in Figure 3, for a perfectly spherical droplet, the surface normal vector is perfectly aligned with the face centroid at every point on the surface, resulting in a dot product of 1. This alignment corresponds to A=0, indicating no deviation from sphericity. However, the detached VOF liquid lump may not achieve a perfect spherical shape due to viscous forces from the surrounding fluid. Therefore, tolerance is given to treat the droplets as spheres. By simplifying the representation of droplet shape, computational resources can be allocated more efficiently, facilitating faster simulations without sacrificing accuracy in modeling droplet behavior. Specifically, two criteria are employed, i.e., if (1) A≤0.2 and (2) there is a volume equivalent diameter $d_{v}\leq 20\;$µm, the VOF liquid lump will be converted into a mass point with the same properties and momentum as the original VOF “droplet” and will be tracked using DPM. It is worth noting that the VOF-to-DPM conversion significantly reduces the computational time. In addition, the mesh refinement surrounding that surface is reset to the original, i.e., the coarser mesh, which ensures that the volume of the surrounding cell is larger than the new point-mass droplet.

#### 2.2.3. DPM

To predict the translation of the DPM droplets, Newton’s 2nd law is employed, i.e.,

(11) $$\frac{d}{dt}\left(m_{d}u_{d,i}\right)=F_{i}^{D}+F_{i}^{L}+F_{i}^{BM}+F_{i}^{G}$$

In Equation (11), the droplet mass $m_{d}$, and components of droplet velocity $u_{d,i}$ are influenced by the drag force $F_{i}^{D}$ [15,16], the Saffman Lift force $F_{i}^{L}$ for spherical droplets in a shearing flow [17], the Brownian Motion-induced force $F_{i}^{BM}$ [18], and the gravitational force $F_{i}^{G}$.

### 2.3. Numerical Setup

Using Ansys Fluent 2024 R2 (Ansys Inc., Canonsburg, PA, USA), the simulations were conducted, which used an explicit VOF scheme with the Geo-Reconstruct method to resolve the interface between the liquid and air phases. The Pressure Implicit with Splitting of Operators (PISOs) algorithm was used for pressure–velocity coupling. The modified body force-weighted pressure discretization scheme was used to balance accuracy, stability, and robustness, particularly when body forces such as surface tension were dominant. The k-ω Shear Stress Transport (SST) turbulence model was used to capture turbulence near the interfaces and within the collision region. Liquid jet inlet conditions were defined using a user-defined function (UDF) to have a velocity profile for fully developed duct flow [19] according to the average inlet velocities listed in Table 1. To investigate how jet inlet velocity, the surface tension of the liquid, and liquid viscosity influence the atomization process, seven cases were created (see Table 1) with different parameter values. The parameter values for Case A were derived from an actual SMI solution and operational condition [20].

**Figure 4:**
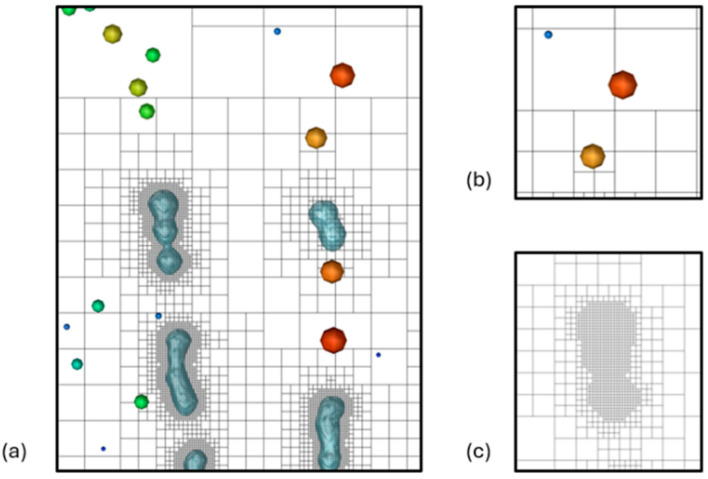
Adaptive mesh refinement of (**a**) combined view of droplet breakup and DPM transition, (**b**) DPM coarsening, and (**c**) refined mesh to resolve liquid interface.

Due to the limitations of the Courant number for this small geometry with high velocities, the time step of these simulations was either 1 × 10^−9^ s or 2 × 10^−9^ s. The Courant–Friedrichs–Lewy (CFL) number limit here is important to ensure that the air–liquid interface is always contained within the refined cells. For the VOF atomization simulations, an end flow time of 4 × 10^−6^ s was selected to ensure that the key atomization characteristics, such as droplet breakup and initial size distribution, were adequately captured. In this study, the primary objective was to observe the atomization process within the mouthpiece and the initial stages of droplet formation rather than tracking every droplet through the exit. Capturing all droplets exiting the mouthpiece would require significantly longer simulation times of up to 20,000 CPU hours compared to 6000 CPU hours for these cases. The simulations were performed on a local Dell Precision T7910 workstation (Intel^®^ Xeon^®^ Processor E5-2683 v4 with dual processors, 32 cores, and 256 GB RAM). Using 32 to 64 threads, the real computational time is approximately 13 days to 30 days for each case. Based on the focus of this study, the chosen end time provided a balance between computational efficiency and capturing the primary breakup characteristics of interest. Adaptive mesh refinement settings for these cases used a minimum cell edge length of 2 × 10^−7^ m. Mesh refinement was performed for each iteration based on the refinement criterion, where cells exhibiting a phase interface curvature exceeding 1 × 10^−12^ m^−1^ were marked for refinement. In contrast, mesh coarsening was performed on refined cells once their curvature decreased below 1 × 10^−14^ m^−1^. Figure 4 represents an example of a section of the mesh refinement near resolved liquid droplets and coarsened near-point-mass DPM.

### 2.4. Model Calibration and Validation

To evaluate the prediction accuracy using the numerical setup for atomization, calibration, and validation were performed by comparing the VOF-to-DPM (i.e., Case A) outputs against benchmark experimental data [20] with the same operational conditions. These simulations were conducted under the nominal operating conditions of the Respimat^®^ SMI to replicate the measured size distributions and assess the accuracy of the VOF-to-DPM in predicting atomized droplet size distributions. The experimental dataset [20] was chosen due to the detailed droplet size and velocity measurements obtained using Phase Doppler Anemometry (PDA). PDA captures droplet characteristics in real time without the influence of secondary processes such as coagulation, evaporation, or condensation. This contrasts with more conventional methods like Andersen Cascade Impactor (ACI) or Next Generation Impactor (NGI) measurements, which can be affected by these additional processes, potentially skewing the size distribution results. Using the PDA-based dataset ensures a more direct comparison of droplet size distributions generated by the Respimat^®^ SMI under realistic operating conditions.

**Figure 5:**
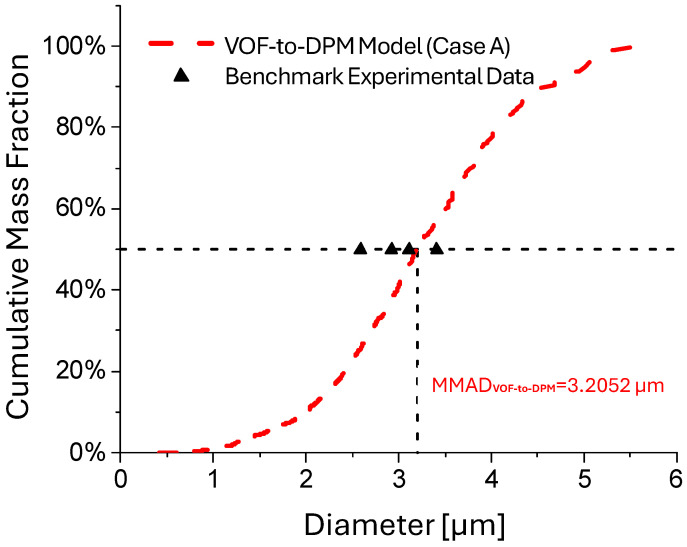
Comparisons of droplet size distributions predicted using the VOF-to-DPM (Case A) and benchmark experimental data [20].

The model calibration involved fine-tuning parameters to align with the experimental droplet size distribution for the base case (i.e., Case A). This step ensured that the VOF-to-DPM accurately captured the atomization and initial droplet formation dynamics under known conditions. While further validation against a range of operating conditions would be optimal beyond the base case comparison only, this validation against limited benchmark experimental data still provides evidence supporting the reliability of the VOF-to-DPM in predicting atomized droplet size distributions. To validate the VOF-to-DPM, the Mass Median Aerodynamic Diameter (MMAD) from the simulation results was compared against the volume-based median diameter ($d_{v50}$) reported in [20]. The comparison is visualized in Figure 5. The uniform droplet density, MMAD and $d_{v50}$ were equivalent, as both represent the 50th percentile of the droplet size distribution by mass or volume, respectively. The present VOF-to-DPM produced an MMAD of 3.205 µm, closely aligning with the $d_{v50}$ values reported in the experimental study [20], which ranged from 2.59 µm to 3.41 µm.

## 3. Results and Discussion

**Figure 6:**
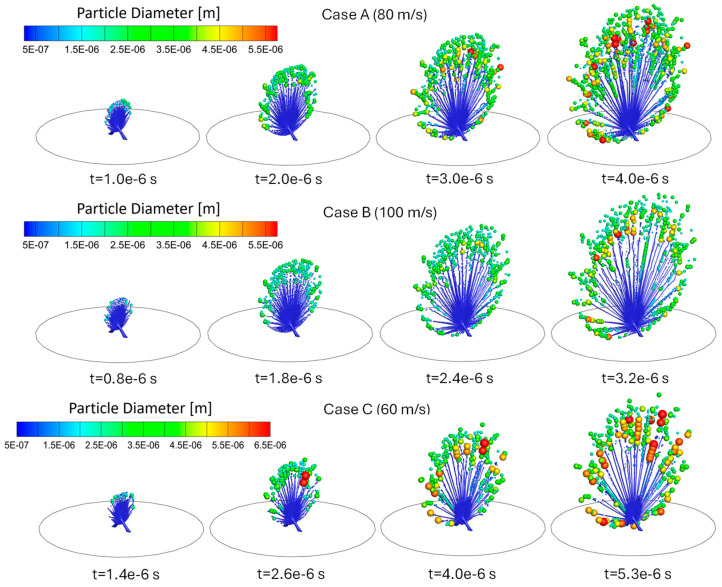
Comparisons of droplet atomization processes among Cases A, B, and C with different colliding jet inlet velocities.

**Figure 7:**
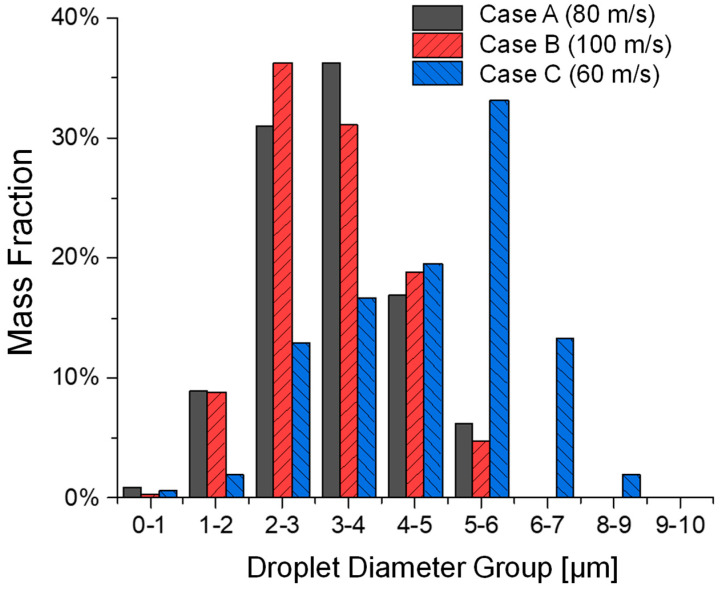
Comparisons of droplet size distributions generated among Cases A, B, and C with different colliding jet inlet velocities.

**Figure 8:**
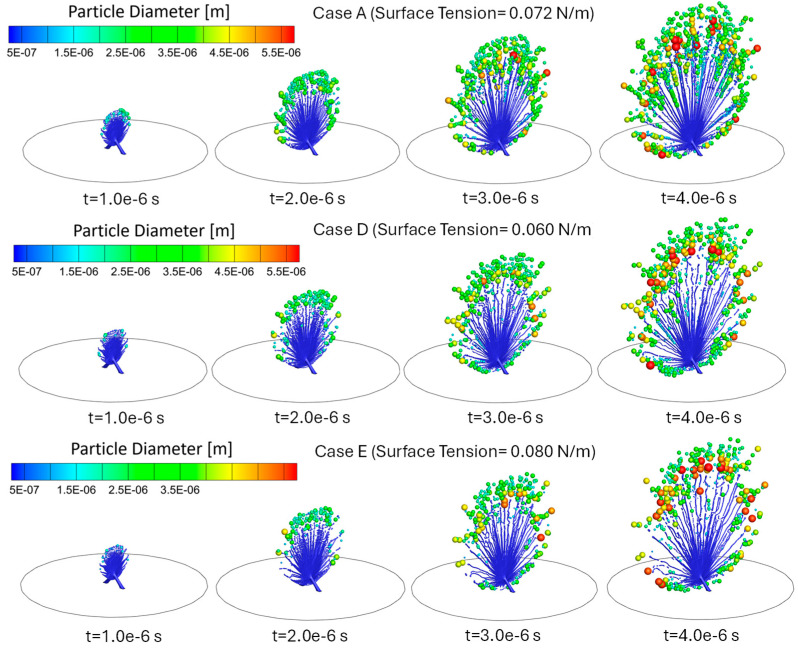
Comparisons of droplet atomization progressions among Cases A, D, and E with different liquid surface tensions.

**Figure 9:**
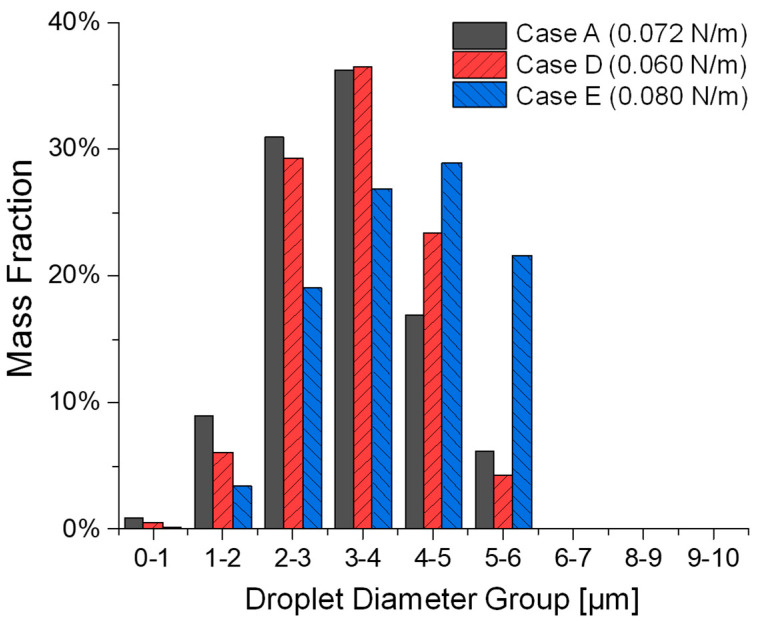
Comparisons of the droplet size distributions generated among Cases A, D, and E with different liquid surface tensions.

**Figure 10:**
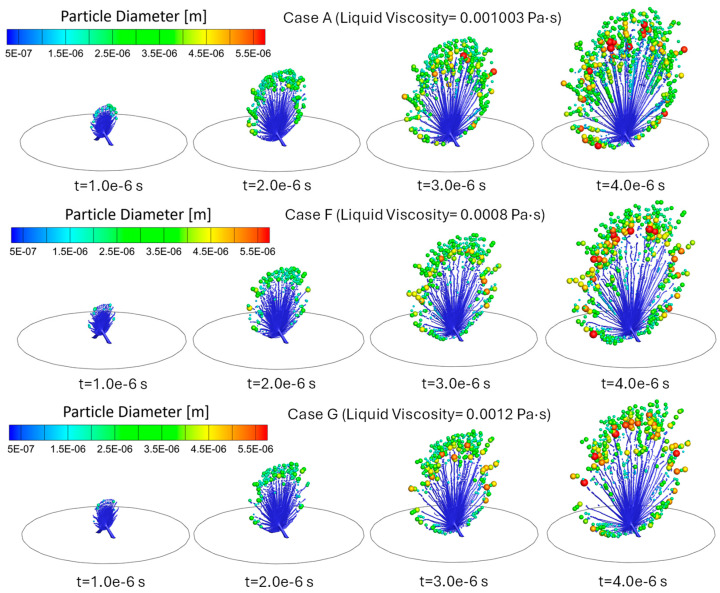
Comparisons of droplet atomization progressions among Cases A, F, and G with different liquid viscosities.

**Figure 11:**
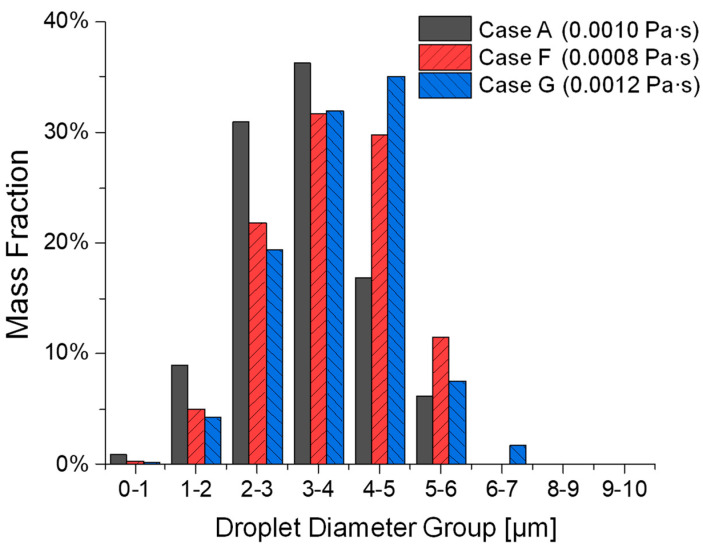
Comparisons of generated droplet size distributions among Cases A, F, and G with different liquid viscosities.

Comparisons of atomization processes and generated droplet size distributions of Cases A to G are shown in Table 2 and Figure 6, Figure 7, Figure 8, Figure 9, Figure 10 and Figure 11. It can be observed that during the collision of the liquid jets, a liquid sheet is formed, which begins to fragment radially into ligaments, which subsequently undergo breakup into droplets. This atomization process occurs in a unique regime of high-liquid Weber ($We_{l}$) numbers (i.e., 640 to 1111, as listed in Table 2) and low-liquid Reynolds numbers ($Re_{l}$) (i.e., 478 to 798). The initial liquid jets exhibit laminar flow, which explains the absence of turbulent breakup and impact waves. Table 2 summarizes the atomized droplet statistics as well as the $We_{l}$ values of the cases. Specifically, the definitions of the spread parameter ($n_{RR}$) for Rosin–Rammler (RR) droplet size distributions and the RR diameter ($d_{m}$) can be found in [21]. Specifically, smaller $d_{m}$ means more fine droplets are generated, which indicates that the atomization process more effectively breaks down the liquid into small droplets. A smaller $n_{RR}$ signifies a narrower distribution, meaning the droplet size generated is more uniform.

For the cases, all simulations used identical numerical setups and time points for comparisons, except for the comparisons between Cases A, B, and C. Indeed, with different colliding jet inlet velocities, the visualizations of atomization processes for Cases A to C were performed at different time points to ensure that the same total mass had entered the domain in each case (see Figure 6).

### 3.1. Influence of Jet Inlet Velocity on Atomization

As shown in Figure 6 and Figure 7, Cases A, B, and C are designed to investigate the effects of different colliding jet inlet velocities while keeping surface tension and viscosity constant. These cases are compared at time stations according to equal injected liquid mass from the colliding jet inlets. Droplet formation occurred more rapidly at higher jet inlet velocities (Case B), producing finer droplets. Case C, with a reduced colliding jet inlet velocity, showed slower droplet formation and larger droplet formation. Case A, the base case, demonstrated intermediate behavior, balancing droplet formation rate and size distribution. The comparison of droplet size distributions in Figure 7 and Table 2 confirms that increased colliding jet inlet velocities enhance atomization, resulting in smaller droplet sizes. Specifically, Case C has a higher MMAD and $d_{10}$ compared to Cases A and B.

Indeed, when velocity increases from 60 m/s in Case C to 80 m/s in Case A and 100 m/s in Case B, both the liquid Weber number ($We_{l}$) and liquid Reynolds number ($Re_{l}$) increase, reflecting a reduced influence of viscous forces relative to the surface tension and inertial force. In Case C, the lowest velocity produces the largest MMAD (i.e., 4.871 µm) because weaker inertial forces allow surface tension to dominate, slowing ligament breakup and generating larger droplets. In Case A, the velocity increases to 80 m/s. The higher inertial forces in Case A promote more efficient ligament breakup, reducing the MMAD to 3.205 µm. In Case B, the highest velocity of 100 m/s leads to the strongest inertial forces and drives even more rapid ligament fragmentation, resulting in the smallest MMAD of 3.125 µm among Cases A to C. The monotonic decrease in MMAD across these cases highlights the dominant role of increasing velocity, as higher $We_{l}$ and $Re_{l}$ overcome surface tension and viscous effects, enhancing atomization efficiency.

The underlying mechanisms discussed above can also be demonstrated from the comparisons of the RR diameter ($d_{m}$) and spread parameter ($n_{RR}$) in Table 2. It can be observed that higher colliding jet inlet velocities (Case B) will generate droplet distributions with smaller $d_{m}$ and $n_{RR}$, indicating a more uniform and finer droplet size distribution. This suggests that increasing the colliding jet inlet velocity enhances the atomization process by breaking the liquid into smaller, more consistent droplets. As a result, the overall efficiency of droplet formation improves. In contrast, the comparison of the total DPM mass atomized in Table 2 demonstrates that with the increase in colliding jet velocity, the total DPM mass atomized decreased.

### 3.2. Influence of Liquid Surface Tension on Atomization

Cases A, D, and E study the impact of varying liquid surface tensions on the atomization in the SMI while keeping the inlet velocity and viscosity consistent. Comparisons can be found in Figure 8 and Figure 9, as well as Table 2. Specifically, reducing surface tension plays a complex dual role in the colliding jet atomization process, i.e., (1) Primary atomization: during the jet breakup stage, it enhances the likelihood of liquid jet breakup into a liquid sheet, increasing the formation of smaller ligaments under the same airflow conditions and shear forces [22]. Consequently, this leads to a higher probability of producing more secondary droplets. (2) Secondary atomization: However, in contrast, during the droplet breakup stage, it also increases the instability of the liquid surface shape, reducing the likelihood of smaller ligaments where surface tension is the dominant force with which to form high-sphericity shapes (i.e., fine droplets) [22]. It can be observed that variations in surface tension significantly influence the dynamics of droplet formation in a complex way, as demonstrated in Cases A, D, and E, where surface tension is systematically varied from 0.06 N/m (Case D) to 0.072 N/m (Case A) and 0.08 N/m (Case E). Despite the inlet velocity being held constant at 80 m/s and viscosity remaining unchanged across all cases, resulting in an identical Reynolds number ($Re_{l}$ = 638.1), the altered balance between capillary and inertial forces is evident in the liquid Weber number ($We_{l}$) and Ohnesorge number ($Oh_{l}$) [23]. Specifically, the lowest surface tension case (Case D) with $\sigma_{l}\;$= 0.06 N/m results in $We_{l}\;$= 853.33 and $Oh_{l}$ = 0.0459. Consequently, Case D exhibits higher MMAD and $d_{10}$ than the baseline case (Case A) (see Figure 9 and Table 2). This increase is attributed to the enhanced likelihood of liquid jet breakup into a liquid sheet, leading to the formation of larger ligaments under the same airflow conditions. However, the reduction in surface tension also increases the instability of the liquid surface shape, which, in turn, decreases the formation of smaller, high-sphericity droplets in regions where surface tension plays a dominant role in fine ligament shape stability. Case A, with an intermediate surface tension $\sigma_{l}$ = 0.072 N/m, yields $We_{l}$ = 711.11 and $Oh_{l}$ = 0.0418. Observed from the simulation results of Case A, a balance between the two roles that surface tension may play on atomization performance produces the smallest MMAD of 3.205 µm among Cases A, D, and E. Case E, characterized by the highest surface tension $\sigma_{l}$ = 0.080 N/m, has $We_{l}$ = 640 and $Oh_{l}$ = 0.0396. The stronger capillary resistance delays ligament breakup, leading to the largest MMAD of 4.013 µm among Cases A, D, and E. The higher surface tension reduces the likelihood of liquid jet breakup into a liquid sheet, thereby limiting the formation of smaller ligaments under the same airflow conditions and shear forces. As a result, the total number of fine droplets decreases, further contributing to the observed increase in MMAD.

Figure 9 and Table 2 also reveal additional complexities in the impact of surface tension on the atomization process. For example, Case D exhibits higher $d_{10}$, $d_{m}$, and $n_{RR}$ as well as lower total DPM mass atomized than Case A, suggesting weakened ligament fragmentation and more significant post-breakup coalescence (see Figure 8). Case E, with the highest surface tension, shows significantly larger $d_{10}$ and $d_{m}$ compared to both Cases A and D. It is very interesting to observe that Case E has the least total DPM mass atomized among Cases A, D, and E. Indeed, the increased surface tension may resist droplet fragmentation, producing larger, more stable droplets with a lower atomization rate (i.e., low total DPM mass atomized) (see Figure 8).

Therefore, Figure 8 and Figure 9, as well as Table 2, highlight the intricate interplay between surface tension, ligament breakup, droplet formation, and atomization efficiency, emphasizing that surface tension reduction does not necessarily lead to a straightforward improvement in droplet formation. Further simulations are necessary to gain deeper insights into these dynamics, incorporating a broader range of surface tension values. Such investigations will help elucidate the relationship between surface tension and atomization performance, providing a more comprehensive understanding of the underlying mechanisms.

### 3.3. Influence of Liquid Viscosity on Atomization

Cases A, F, and G focus on the effect of different liquid viscosities on atomization while maintaining the same inlet velocity ($U_{in}$ = 80 m/s) and surface tension ($\sigma_{l}$ = 0.072 N/m). Specifically, the viscosity increases from $\mu_{l}$= 0.0008 Pa·s in Case F to $\mu_{l}$ = 0.001003 Pa·s in Case A and $\mu_{l}$ = 0.0012 Pa·s in Case G. Although Figure 10 does not show significant observable differences in the atomization process, comparisons in Figure 11 and Table 2 indicate some non-monotonic trends between the atomization effectiveness in generating fine droplets vs. the increase in liquid viscosity.

Specifically, it can be seen from Figure 11 and the comparison of $d_{10}$, $d_{m}$, MMAD, and $n_{RR}$ in Table 2 that Case F (lower viscosity) has higher $d_{10}$, $d_{m}$, and $n_{RR}$ than the baseline Case A. Such a comparison indicates that Case F shows less effective atomization in the generation of finer droplets than Case A. The higher $d_{10}$ and MMAD may suggest that while atomization is easier with a lower viscosity, the distribution of droplet sizes results in a higher median. A possible explanation for this is that lower viscosity weakens ligament cohesion, making ligaments more susceptible to rapid disintegration before they can stretch into finer filaments, leading to a less uniform droplet size distribution [24,25]. Additionally, reduced viscosity diminishes resistance to droplet merging, increasing the likelihood of coalescence in high-density spray regions [25]. As a result, while lower viscosity theoretically enhances atomization, the observed increase in $d_{10}$ and MMAD indicates that the droplet size distribution favors a higher median diameter, ultimately reducing overall atomization efficiency. Comparisons in Figure 11 and Table 2 also show that Case G, with the highest liquid viscosity, generated droplet size distributions with higher $d_{10}$, $d_{m}$, MMAD and $n_{RR}$ than Case A. This could be attributed to the increased liquid viscosity, which enhances resistance to deformation and stretching, suppressing the formation of smaller ligaments by requiring higher breakup energy and allowing capillary forces to dominate over inertial forces. As a result, larger droplets are formed, leading to lower atomization efficiency. These results demonstrate that the increasing viscosity and the resultant increase in $Oh_{l}$ suppresses ligament breakup, leading to larger droplet sizes [25]. As the baseline case, with higher $\mu_{l}$ than Case F and lower $\mu_{l}$ than Case G, Case A ($\mu_{l}$ = 0.001003 Pa·s) has a moderate Ohnesorge number ($Oh_{l}$ = 0.0418) in this comparison, indicating a balance between the dual roles of viscosity on atomization. This balance allows efficient ligament breakup while limiting excessive viscous resistance, resulting in the smallest MMAD of 3.205 µm among Cases A, F, and G. Although $Oh_{l}$ of Case A is between that of Cases F and G, the resulting MMAD is smaller than expected because the balance of forces promotes efficient fragmentation, yielding a narrower distribution of smaller droplets.

Both Cases F and G demonstrate lower total DPM mass atomization compared to Case A. However, Case F exhibits a higher total DPM mass atomization than Case G. This indicates that while lower viscosity facilitates atomization, it may also promote increased droplet interactions and potential coalescence, preventing the ligament volume from becoming sufficiently small to convert into DPM. In contrast, the higher viscosity in Case G inhibits the atomization process, resulting in fewer droplets and a lower mass of atomized medication.

## 4. Conclusions

Employing a validated VOF-to-DPM, this study highlights the complex interplay between colliding jet inlet velocity, surface tension, and liquid viscosity, influencing the atomization process in Respimat^®^ SMI. Key conclusions based on the VOF-to-DPM simulation results include the following:

- Increased colliding jet inlet velocities (i.e., Case C → A → B) improve atomization efficiency by enhancing ligament fragmentation, producing finer droplets with narrower size distributions. However, higher velocities (i.e., Case C → A → B) reduce the total atomized droplet mass, potentially reducing the emitted dose.
- Surface tension exhibits the non-monotonic effect on atomization. Lower surface tension (i.e., Case D vs. Case A and Case E) promotes droplet breakup but may also increase post-breakup coalescence, leading to larger median sizes. Conversely, higher surface tension (i.e., Case E vs. Case A and Case D) resists breakup, resulting in fewer, larger, and more stable droplets.
- Lower viscosity (i.e., Case F vs. Case A and Case G) aids atomization by reducing resistance to ligament breakup but can lead to coalescence, increasing droplet size. Higher viscosity (i.e., Case G vs. Case A and Case F) inhibits atomization, producing fewer, larger droplets with a lower total atomized mass.

The findings listed above underscore the importance of balancing the three parameters to achieve optimal droplet size distributions for efficient pulmonary drug delivery.

## 5. Limitations of This Study and Future Work

The limitations of this study include the following points:

- The non-monotonic trends observed in surface tension and the effects of viscosity require further exploration to fully understand the underlying mechanisms, including secondary breakup and droplet coalescence.
- This study focuses on the initial atomization process without considering downstream droplet transport, evaporation, or deposition in realistic airway geometries.
- To address the limitations mentioned above, future work should attempt the following:
- Extending the study with more surface tension and liquid viscosity properties can generate more insights into the underlying mechanisms leading to non-monotonic trends.
- Investigating the effects of nozzle geometry, impingement angles, and additional operational parameters on atomization dynamics, as well as exploring the role of drug formulation properties, such as surfactants and active ingredient concentrations, on atomization and delivery efficiency.
- Incorporating user-defined functions (UDFs) for the further customization of the VOF-to-DPM to capture droplet interactions, secondary breakup, and coalescence during transport.
- Couple atomization simulations with downstream airflow and deposition models to evaluate the performance of SMIs in realistic respiratory systems.

## Figures and Tables

**Table 1:** Summary of case names and adjusted parameters.

Case	Average Jet Inlet Velocity ($U_{in}$) [m/s]	Surface Tension ($\sigma_{l}$) [N/m]	Liquid Viscosity ($\mu_{l}$) [Pa·s]
A	80	0.072	0.001003
B	100	0.072	0.001003
C	60	0.072	0.001003
D	80	0.06	0.001003
E	80	0.08	0.001003
F	80	0.072	0.0008
G	80	0.072	0.0012

**Table 2:** Comparison of liquid phase Weber numbers, Reynolds numbers, Ohnesorge numbers, arithmetic mean diameters, total DPM masses atomized, MMAD, RR diameters, and spread parameters for Cases A to G.

Case	Liquid Weber Number ($We_{l}$)	Liquid Reynolds Number ($Re_{l}$)		Ohnesorge Number ($Oh_{l}$)
A	711	638.1		0.0418
B	1111	797.6		0.0418
C	400	478.6		0.0418
D	853	638.1		0.0458
E	640	638.1		0.0396
F	711	800.0		0.0333
G	711	533.3		0.0500
**Case**	** Arithmetic Mean Diameter** **(** $d_{10}$ **) [m]**	**Total DPM Mass Atomized ** **[kg]**		**MMAD ** ** [m]**
A	1.89 × 10^−6^	8.37 × 10^−12^		3.21 × 10^−6^
B	2.10 × 10^−6^	6.54 × 10^−12^		3.13 × 10^−6^
C	2.18 × 10^−6^	9.36 × 10^−12^		4.87 × 10^−6^
D	2.16 × 10^−6^	6.78 × 10^−12^		3.38 × 10^−6^
E	2.52 × 10^−6^	6.21 × 10^−12^		4.01 × 10^−6^
F	2.33 × 10^−6^	7.67 × 10^−12^		3.64 × 10^−6^
G	2.51 × 10^−6^	6.59 × 10^−12^		3.76 × 10^−6^
**Case**	**RR Diameter** **(** $d_{m}$ **) [m]**		**Spread Parameter** **(** $n_{RR}$ **)**	
A	3.54 × 10^−6^		3.68	
B	3.47 × 10^−6^		3.62	
C	5.34 × 10^−6^		4.0	
D	3.72 × 10^−6^		3.92	
E	4.37 × 10^−6^		3.89	
F	4.11 × 10^−6^		3.78	
G	4.11 × 10^−6^		4.19	

## Data Availability

The data that support the findings of this study are available from the corresponding author upon reasonable request.

## Abbreviations

The following abbreviations are used in this manuscript:

ACI	Andersen Cascade Impactor
AMR	Adaptive mesh refinement
CFL	Courant–Friedrichs–Lewy
CFD	Computational fluid dynamics
DPM	Discrete Phase Model
MMAD	Mass Median Aerodynamic Diameter
NGI	Next Generation Impactor
Oh	Ohnesorge number
PDA	Phase Doppler Anemometry
PISO	Pressure-Implicit with Splitting of Operators
PLIC	Piecewise linear interface reconstruction
Re	Reynolds number
RR	Rosin–Rammler
SMI	Soft Mist inhaler
SST	Shear Stress Transport
UDF	User-Defined Function
VOF	Volume of Fluid
We	Weber number
